# A Model for Osteonecrosis of the Jaw with Zoledronate Treatment following Repeated Major Trauma

**DOI:** 10.1371/journal.pone.0132520

**Published:** 2015-07-17

**Authors:** R. Nicole Howie, James L. Borke, Zoya Kurago, Asma Daoudi, James Cray, Ibrahim E. Zakhary, Tara L. Brown, J. Nathan Raley, Loan T. Tran, Regina Messer, Fardous Medani, Mohammed E. Elsalanty

**Affiliations:** 1 College of Dental Medicine, Georgia Regents University, Augusta, Georgia, United States of America; 2 College of Dental Medicine, Western University of Health Sciences, Pomona, California, United States of America; 3 College of Science and Mathematics, Georgia Regents University, Augusta, Georgia, United States of America; 4 College of Dental Medicine, Medical University of South Carolina, Charleston, South Carolina, United States of America; 5 School of Dentistry, University of Detroit-Mercy, Detroit, Michigan, United States of America; Faculté de médecine de Nantes, FRANCE

## Abstract

This study aims to develop a reproducible rat model for post-traumatic bisphosphonate-related osteonecrosis of the jaw (BRONJ). In our previous studies using dental extraction as an inducing factor, only 30% - 60% of zoledronate-treated animals fulfilled the definition of clinical BRONJ. We modified the zoledronate regimen and introduced repeated surgical extraction to illicit quantifiable BRONJ in all animals. Eighty retired-breeder female Sprague-Dawley rats were divided between the treatment (IV zoledronate; 80 μg/kg/week for 13 weeks) and control (saline) groups. On week 13, the left mandibular first molar was surgically extracted, followed by the second molar a week later. Animals were euthanized at 1-week, 2-weeks, and 8-weeks following extraction. The occurrence and severity of BRONJ were scored in each animal based on gross and MicroCT analysis. Parameters of bone formation and osteoclast functions at the extraction site were compared between groups. All zoledronate-treated animals developed a severe case of BRONJ that fulfilled the clinical definition of the condition in humans. Osteoclast attachment continued to be defective eight weeks after stopping the treatment. There were no signs of kidney or liver toxicity. Our data confirmed that repeated surgical extraction (major trauma) by itself consistently precipitated massive bone necrosis in ZA-treated animals, eliminating the need to induce pre-existing infection or comorbidity. These results will be the basis for further studies examining the *in-vivo* pathogenesis and prevention of BRONJ.

## Introduction

Intravenous bisphosphonates has been linked to osteonecrosis of the jawbone, a condition termed bisphosphonate-related osteonecrosis of the jaw (BRONJ). Studies to date have not identified a direct cause for BRONJ *in vivo*. It is yet to be demonstrated that therapeutic regimens of intravenous bisphosphonates consistently induce BRONJ in animals, irrespective of pre-existing comorbidity or local infection. In their 2014 status update, the American Association of Oral and Maxillofacial Surgeons (AAMOS) emphasized the need for reliable animal models that can be utilized to test potential treatment and prevention protocols.[1]

Although many animal models have been described in the literature (Table 1) such translational models are lacking. Most existing models involved the induction of either an independent infection/inflammation, such as periodontitis,[2,3] or periapical disease;[4,5] or a systemic comorbidity, such as vitamin D deficiency,[6] or dexamethasone therapy.[7] These models are useful for studying BRONJ in the context of the pre-existing co-morbidity. On the other hand, clinical BRONJ mostly followed invasive dental procedures,[1,8] suggesting that a key trigger of BRONJ likely involves a drug-induced compromise in the bone response to invasive trauma. Even though the underlying indication for dental extraction in these patients may have been infection, BRONJ did not manifest until after extraction in most cases.[1] For a direct *in-vivo* mechanism to be identified, it is yet unclear whether invasive trauma by itself is sufficient to precipitate BRONJ in bisphosphonate-treated individuals.[1]

**Table 1:** Summary of Previous BRONJ Animal Models.

Study (year)	Species	Drug	Dose	Inducing Factor(s)	Comments
Ersan et al. (2014) [33]	Rat	0.2 mg/kg Za	3 per week for 6 weeks	1^st^ mandibular molar extracted	0% clinical presentation of BRONJ
Aghaloo et al. (2014)[4]	Mouse	10mg/kg RANKL-Fc	3 per week for 3 weeks	1^st^ and 2^nd^ mandibular molars extracted coupled with drilling	30% clinical/radiographic incident rate
Guevarra et al. 2013[9]	Rat	20ug/kg Za	2 doses, 4 weeks apart	1^st^ mandibular molar extracted	30% clinical incident rate
Kang et al. (2013)	Mouse	200ug/kg Za	3 times	Periapical lesion	30% clinical presentation; no radiographic evidence
Conte Neto et al. (2013)[15]	Rat	1.0mg/kg Aln	1 per day for 60 days	1^st^ mandibular molar extracted	Sacrificed at 4 weeks
Abtahi et al. (2013)[35,39]	Rat	200ug/kg Za200ug/kg Aln	Daily for 14 days	Implant or 1^st^ maxillary molar extracted	Sacrificed at 2 weeks
Pautke et al. (2012)[36]	Minipig	0.05mg/kg Za	1 per week for 10 weeks	3 maxillary and 3 mandibular molars extracted	80% incident rate with a massive traumatic injury induced
Marino et al. (2012)[10]	Rat	20ug/kg Za	2 doses, 4 weeks apart	1^st^ mandibular molar extracted	60% clinical incident rate but with no radiographic or histological evidence
Aguirre et al. (2012)[3]	Rat	15ug/kg Aln8ug/kg Za80ug/kg Za	1 per month for 6, 12, or 18 weeks	Periodontitis	11% clinical incident rate in controls with no radiographic or histological evidence
Allen et al. (2011)[30]	Dog	0.06mg/kg Za	Every 2 weeks for 8 months	2 mandibular extractions 1 month apart	0% clinical, radiographic, or histological incident rate
Cankaya et al. (2011)[37]	Rat	0.1mg/kg Za	3 per week for 10 weeks	All left mandibular molars or all left maxillary molars extracted	Sacrificed at 4 weeks
Ali-Erdem et al. (2011)[38]	Rat	7.5ug/kg Za	1 per week for 4 weeks	1^st^ and 2^nd^ maxillary and mandibular molars extracted	60% incident rate in treatment group; 30% in control group. Sacrificed at 4 weeks
Aghaloo et al. (2011)[2]	Rat	66ug/kg Za	3 per week for 15 weeks	Periodontal disease	0% clinical presentation; 32% radiographic incident rate; 50% treatment histological incident rate, 10% in control
Kikuiri et al. (2010)[40]	Mouse	125ug/ka Za	2 per week for 2 weeks	1^st^ maxillary molar extracted	Sacrificed at 7 weeks and only had a 10% incident rate
Biasotto et al. (2010)[41]	Rat	0.04mg/kg Za	1 per week for 5 weeks	Maxillary molar extraction	100% incident rate but in a small sample size (5 animals)
Sonis et al. (2009)[7]	Rat	7.5ug/kg Za	1, 2, or 3 doses	1^st^ and 2^nd^ maxillary and mandibular molars extracted	60% incident rate

In our previous studies using a single dental extraction as the inducing factor for BRONJ, only 30%- 60% of treated animals fulfilled the BRONJ definition. Even though clear differences in bone vitality were evident between treated and untreated groups, the variability in BRONJ occurrence and features between animals jeopardized the reliability of the model for future translational studies.[9,10] The next step was to modify the zoledronate regimen, simulating long-term therapy, and use a more significant trauma to consistently illicit BRONJ in all animals, without the need to induce independent local and/or systemic comorbidity.

Zoledronic acid (zoledronate; ZA) is the most potent bisphosphonate.[11] It also carries the highest incidence of BRONJ.[8] Cancer patients receive an intravenous infusion of 4mg of zoledronate over 15 minutes every 3–4 weeks.[12,13] The overall prevalence of BRONJ with zoledronate is around 7.7%, if the therapy continues for 37 to 48 months,[8,14] and is expected to increase as more long-term results emerge. Osteonecrosis involved the mandible in 65%, the maxilla in 26%, and both sites in 9% of the reported cases.[8] Previous models that utilized trauma as the sole morbidity utilized very high doses of bisphosphonates that were outside the therapeutic margin for humans.[15] Furthermore, all previous studies, including ours, focused their analysis on the collective trends in the study population, making it difficult to predict the likelihood of creating the complete picture of BRONJ in each animal.

The aim of the current study is to test a BRONJ model with the following characteristics: 1) demonstrates consistent occurrence of the full features of BRONJ in bisphosphonate-treated animals, using a clinically-relevant bisphosphonate dose and a precipitating factor that can be calibrated (repeated surgical extractions); 2) excludes the systemic toxic effects of the bisphosphonate regimen on the liver and kidney; and 3) provides a quantitative BRONJ analysis. We hypothesized that repeated surgical extraction (major trauma) would be sufficient to induce consistent, severe form of BRONJ in rats on long-term zoledronate therapy, without the need to induce pre-existing dental and/or systemic pathology. This step is necessary for subsequent studies to determine if a causal relationship exists between bisphosphonates, specifically zoledronate, and post-traumatic osteonecrosis *in-vivo*, as well as test possible prevention strategies.

## Animals and Methods

### Animals Groups

The experimental procedures were reviewed and approved by the Institutional Animal Care and Use Committee (IACUC) at Georgia Regents University (Protocol #2012–0496; Date: 10/25/2012). The study involved 80 female Sprague-Dawley rats (age 10–12 months-old). At this age, the size of the animals allowed the optimization of the surgical procedure. The study involved female rats to allow comparisons with our previous results using the osteoporosis-dose of zoledronate. Animals were randomly assigned into two groups (40 animals-each): control and zoledronate (ZA)-treated. Each animal received a weekly 0.3ml intravenous injection via the tail vein for 13 weeks of either ZA (Enzo LifeSciences, Farmingdale, NY, USA; 80 μg/kg body weight in phosphate-buffered saline), or saline only, followed by molar extraction on the left side. Injections were carried out under isoflurane anesthesia. The study was done on two phases; each included half the control and treated animals. The study groups at 1-week, 2-weeks, and 8-weeks post-extraction included: the control-extraction site, control-contralateral side, ZA-treated extraction site, ZA-treated-contralateral side.

### Dental Extraction

Two experienced surgeons, blinded to the animal groups, carried out the surgical extractions. Extraction of the left mandibular left first molar was performed at week 13 of the study using Adson surgical forceps. Extractions were done under anesthesia using intraperitoneal injections of ketamine (100 mg/mL) and xylazine (20 mg/mL). At week 14, the mandibular left second molar was extracted in the same way in each animal in the 8-week groups (40 animals). At the time of each extraction, the extraction socket was cleaned using a 1.0 mm round bur at 15,000 RPM to remove any remaining root fragments. The use of the bur aimed at standardizing the extraction defect. The procedure would therefore be comparable to surgical, as opposed to simple, dental extraction in human patients. Analgesia with buprenorphine was made available post-operatively but was not used. Animals were closely observed for signs of bleeding, discomfort, or lack of feeding and were fed crushed pellets for 3 days after extractions.

### Euthanasia and Sample Collection

In each group, 10 animals were euthanized after 1 week and 10 animals after 2 weeks following the first extraction and 20 animals after 8 weeks following the second extraction. Euthanasia was carried out by an overdose of CO_2_ in a closed plastic chamber, followed by exsanguination. This method meets the recommendations of The American Veterinary Medical Association. Blood was withdrawn for serum analysis via an intracardiac catheter. Immediately following euthanasia, the animals were carefully dissected to collect the mandible, liver, and kidneys. All samples were coded for subsequent blinded analysis.

### Criteria for Osteonecrosis

For each animal in 8-week group (n = 40), the incidence and severity of BRONJ was analyzed as follows:

1. Gross: Persistent failure of mucosal coverage, with exposed necrotic bone, at week-8
2. Radiographic: Sequestration of the alveolar bone by micro-CT analysis
3. Based on criteria 1 and 2, each animal was given a categorical (0–4) score for the severity of osteonecrosis:
  - Grade 0: Complete coverage of the mucosa at the extraction site and normal healing patterns in micro-CT
  - Grade 1: Bone exposure at the extraction site at week-8 by gross examination, without micro-CT evidence of sequestration
  - Grade 2: Bone exposure plus radiographic evidence of sequestration at the extraction site by micro-CT in two planes
  - Grade 3: Bone exposure plus radiographic evidence of complete sequestration of bone at the extraction site by micro-CT in three planes.
  - Grade 4: Same as grade-3, plus sequestration of the entire alveolar ridge
4. Lack of bone healing at the socket site by bone dynamic analysis
5. Confirmation of bone tissue necrosis by histomorphometry
6. Deficient osteoclast functions by TRAP staining

### Clinical Images and Micro-CT

Intraoral photographs were taken every two weeks during the follow-up period. After euthanasia, the extraction site in each animal was photographed to identify any persistent exposure of necrotic bone. The mandibles from the 8-week group were scanned by microCT as described previously.[16] In brief, specimens were scanned using an *ex vivo* microCT system (Skyscan 1174; Skyscan, Aartlesaar, Belgium). Each sample was scanned in air using a 0.25-mm aluminum filter, 15.9μ isotropic voxels, 1600ms integration time, 0.5° rotation step, and frame averaging of 3. For 3-D reconstruction (NRecon software, Skyscan), the grey scale was set from 80 to 200. We then examined the reconstructed images to identify evidence and extent of three-dimensional sequestration of the alveolar bone. Sequestered bone was differentiated from root tips and chipped bone upon sizes and shapes of the bone fragments, whether the pulp spaces could be seen, and if the extraction sites could be seen within the bone fragments.

### Histology

The segments of the mandibles containing extraction sites, as well as the contralateral mandibular sites, from the 1-week and 8-week group animals were dissected and coded for blinded analysis. The soft tissues were removed and the bone was preserved in 10% phosphate-buffered formalin (1:10 by volume) for 48 hours. This was followed by demineralization with EDTA (17%) for 3 weeks, during which the containers were placed on a continuous shaker at 4°C; and EDTA was changed every 2–3 days. Demineralization was confirmed by soft x-ray and demineralized samples were processed overnight using a semi-enclosed Bench-top Tissue Processor (Leica Microsystems, Buffalo Grove, IL). Before embedding in paraffin, each sample was sectioned in a coronal plane through the extraction site. Sections were cut at 5 microns and stained with hematoxylin and eosin using a standard protocol. Three 20x fields where examined in each section focusing on the intact buccal, lingual, and basal walls of the socket (Zeiss AxioImager M2, Carl Zeiss Microscopy GmbH, Gena, Germany). The degree of alveolar bone necrosis was quantified as the ratio between empty osteocyte lacunae and the total number of lacunae in the field. The extraction sites were compared between the control and ZA-treated animals. Also, the un-operated sites were compared between the two groups. Sequestrated and separated bone fragments were not included in the analysis. Within each micrograph, osteonecrosis was quantified as the ratio between empty osteocyte lacunae to the total number of lacunae.

### Evaluation of Bacterial Colonization

We compared the rate of bacterial colonization between the control and ZA-treated animals in the 1-week samples. The rationale was to detect early difference in bacterial colonization between the treated and control animals that may contribute to the induction of BRONJ. After demineralization, coronal sections of the extraction sites from week-1 animals (10 controls; 10 ZA-treated) were embedded in paraffin and serial sections were cut at 5μ thickness. Analysis was carried out blindly. For localization of gram-negative and gram-positive bacteria, the sections were stained using a modified Brown and Brenn method.[17] First, sections were deparaffinized, hydrated with distilled water, and washed with 1% Crystal Violet solution and Gram’s Iodine solution respectively for one minute each. Slides were rinsed after each stain to remove excess dye. This was followed by decolorization using acetone then immediately rinsing in tap water. Then, the slides were treated with 0.25% Basic Fuchsin for 3 minutes, then rinsed, and then dipped twice in acetone solution. Then, the slides were treated with Picric acid-acetone for 10 seconds, then twice with absolute acetone for a total of 4 seconds. This was followed by two dips in xylene solution. Finally, the slide was mounted and covered. Photomicrographs of these slides were compared at 20x and 100x. Gram-positive bacteria would stain blue, gram-negative would stain pink, nuclei should stain red, and the background should stain yellow. Controls micrographs were taken of *E*.*coli* and *S*. *Aureus* live cultures.

### Bone Dynamics Analysis

The 2-week animals (10 control and 10 ZA-treated) were coded for blinded bone dynamics analysis. The aim of this step was to detect changes in bone formation dynamics between the treated and control animals at the early phase of bone regeneration. Each animal received two intraperitoneal calcein injections (Sigma Aldrich, Inc.; Catalog #: C0875 20mg calcein/Kg in saline solution; 6mg in 0.2 ml/ rat) three days apart and was sacrificed 48 hours following the second injection. The bone segments containing the extraction sites were preserved in 70% alcohol. Samples were dehydrated in increasing concentrations of alcohol, followed by Xylene, and then embedded in methylmethacrylate resin. After complete polymerization, the area of interest was brought closer to the surface by preliminary grinding, and the opposite side of the block was mounted on a slide using Technovit 4000 (*Exakt Technologies*, *Inc*., *Oklahoma City*, *OK*). One sagittally-oriented section (about 100 microns thick) per animal was cut from each specimen using the water-cooled Exakt Diamond Saw (Exakt Technologies, Inc., Oklahoma City, OK). The sections were ground to about 20–30μm with the Exakt Grinding System (*Exakt Technologies*, *Inc*., *Oklahoma City*, *OK*) and polished with 4000 grit sandpaper and Novus Polish. Three fields were analyzed in each section. The mineral apposition rate (MAR, μ/day), defined as the distance between the midpoints of the double label (dL) divided by the number of days between calcein injections, was measured. The mineral formation rate (MFR/B.Pm, μ^3^/μ^2^/day) was calculated as an indication of the amount of newly mineralized bone MFR = MAR*((dL.Pm+sL.Pm/2)*B.Pm)/B.Pm), where dL.pM is the double label perimeter, sL.Pm is the single label perimeter, and B.Pm is the entire bone perimeter.[18]

### Osteoclast Parameters

Osteoclasts were counted on slides assayed for tartrate-resistant acid phosphatase (TRAP) activity as TRAP-positive multinucleated cells. The aim of this step was to detect differences in bone resorption parameters between the treated and control animals, as well as before and after the establishment of BRONJ. Coded slides were deparaffinized in xylene, hydrated in serial concentrations of ethanol, and incubated in a solution containing diazotized fast garnet, napthol AS-BI phosphate, acetate, and tartrate solution from the Acid Phosphatase, Leukocyte (TRAP) kit (Sigma-Aldrich) as described previously.[19] Osteoclasts were identified as TRAP-positive cells that contained 3 or more nuclei and were counted utilizing Image-J software (http://imagej.nih.gov/ij/). Osteoclasts were counted at the extraction site in 3 randomly chosen fields at 20x from 1-week (n = 10) and 8-week (n = 20) groups, evenly split between control and treated groups and analyzed for osteoclast number per bone surface perimeter (N.Oc/B.Pm, μ^-1^) and osteoclast surface (Oc.Pm/B.Pm, %; calculated as a percentage of osteoclast perimeter (Oc.Pm, μ) to bone surface perimeter (B.Pm, μ)). Osteoclast surface would correct for the difference in the size of osteoclasts that might skew the measurement of osteoclast number. The nomenclature used in this report followed the 2012 Update of the Report of the ASBMR Histomorphometry Nomenclature Committee.[18]

### Histopathology of the Kidneys and Liver

A blinded, pathologist (ZK) assessed the liver and kidney sections for evidence of drug toxicity. Kidneys were assessed at 1, 2 and 8 weeks after dental extractions (5 samples per group). Livers were assessed similarly at 1 and 8 weeks after extraction. Entire hematoxylin and eosin-stained sections were examined microscopically and the features tabulated. For photomicrographs, one case per sample from each group was selected at random.

### Liver and Kidney Functions

To further assess potential liver and kidney injury due to long-term zoledronate treatment, we measured the serum levels of alanine transaminase (ALT) and blood urea nitrogen at weeks 1, 2, and 8 after extraction. We followed the manufacturers’ instructions for the following kits: ALT Assay Sigma-Aldrich: Cat # MAK052-1KT (lot: b8h120752v) and Urea Assay Kit Abcam: Cat # ab83362 (lot GR138519-5)

### Statistical Analysis

Animals were randomly assigned to the control or treated groups. At surgery, the surgeon was not informed as to which group the animal belonged. The animals were done on two completely separate stages (each has 20 control and 20 ZA-treated rats) to ensure that factors such as lab conditions, surgical room conditions, operator, animal patch, etc did not affect the healing. At necropsy, all samples were coded and from that point on each sample was identified only by its code. The sample allocation to different tests, sample processing, and data collection were completely blinded. Based on an effect size of 1.64, calculated from previous studies,[2] a minimum sample size of 10 animals was determined to yield a study power of 0.96 (G*Power v.3.1) for comparing means from independent groups.[20]

Un-coded data were tabulated per group for statistical analysis. Statistical analysis was done using SPSS Statistics software for Windows (v. 20; IBM,Corp, New York, USA). Values were represented as mean±SD. Normality and equal variances assumptions for each test were evaluated using the Shapiro-Wilk test and Levene’s Test, respectively. A one-way ANOVA test with significance defined as p < 0.05 and Bonferroni post-hoc comparison was used to compare multiple groups. Comparison of BRONJ severity score was done using chi^2^ analysis with a Fisher’s exact correction (Pearson Chi-Square F = 39). For histolomorphometric analysis, a natural log transformation and welsh correction was applied to the data to correct for violation of the normality and equal variances assumptions, respectively. Dynamic parameters were analyzed using by a two-tailed student’s t-test with unequal variances. Osteoclast surface was analyzed using a one-way ANOVA with a Welsh correction. The percentage of attached osteoclasts was analyzed using a rank transformation and a two-way ANOVA. Changes in animal weight were compared using repeated-measures ANOVA.

## Results

### Gross Evaluation

**Fig 1:**
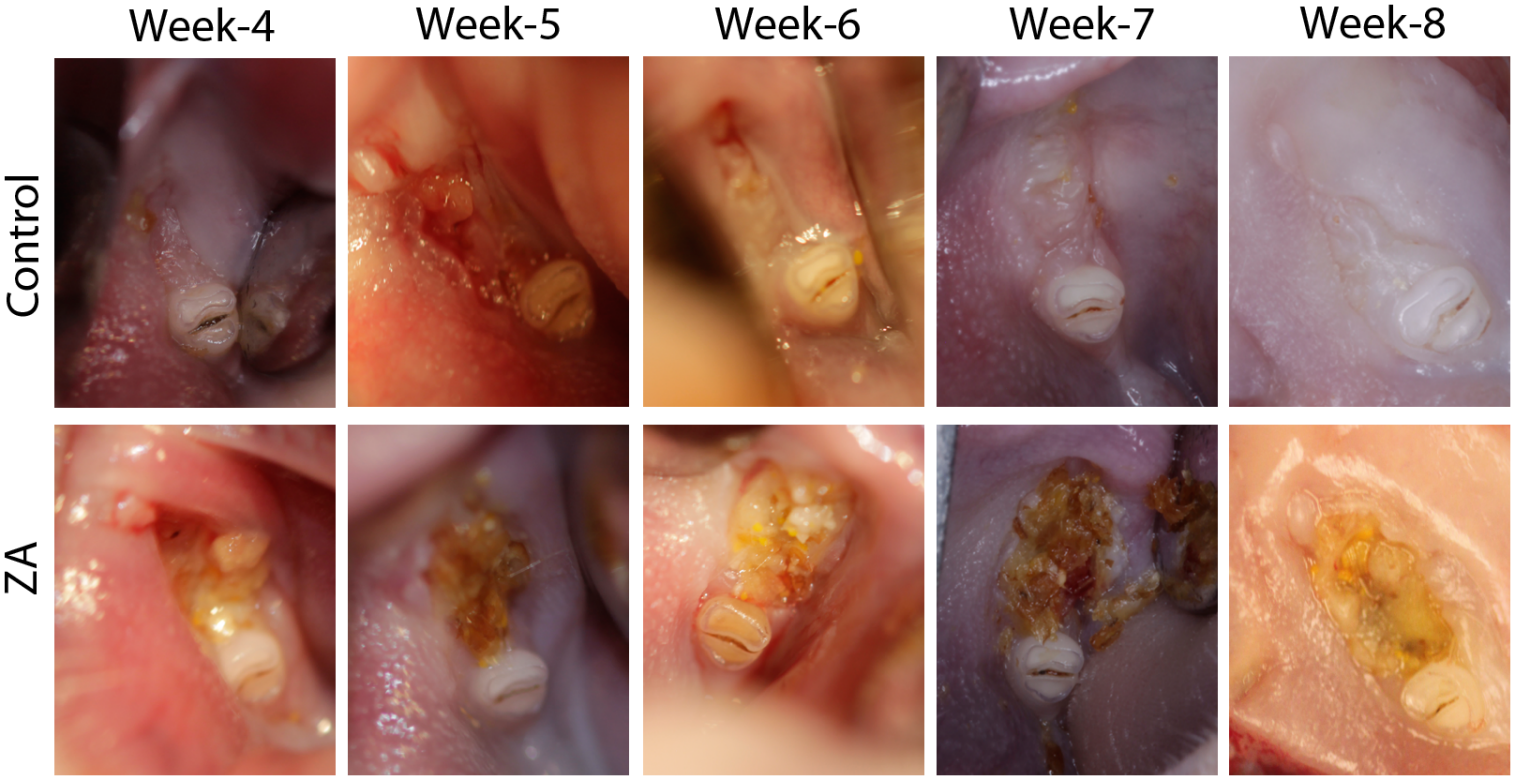
Intraoral images of extraction sites in control vs. ZA-treated rats, showing persistent exposure of necrotic bone in the ZA-treated animals 8 weeks after extraction. Untreated animals showed complete healing.

As early as 4 weeks after extraction, the control animals showed healthy, complete mucosal covering of the extraction sites with no inflammation, discoloration, or loose bone fragments evident (Fig 1 and S1 Fig). Remaining root tips were evident at the extraction sites in a few controls, with signs of localized mucosal inflammation. The underlying bone and surrounding mucosa appeared normal in these animals. All ZA-treated animals, however, exhibited progressive mucosal dehiscence, exposing underlying surfaces of desiccated, dark-colored bone at the extraction sites (Fig 1 and S2 Fig).

### Micro-CT

**Fig 2:**
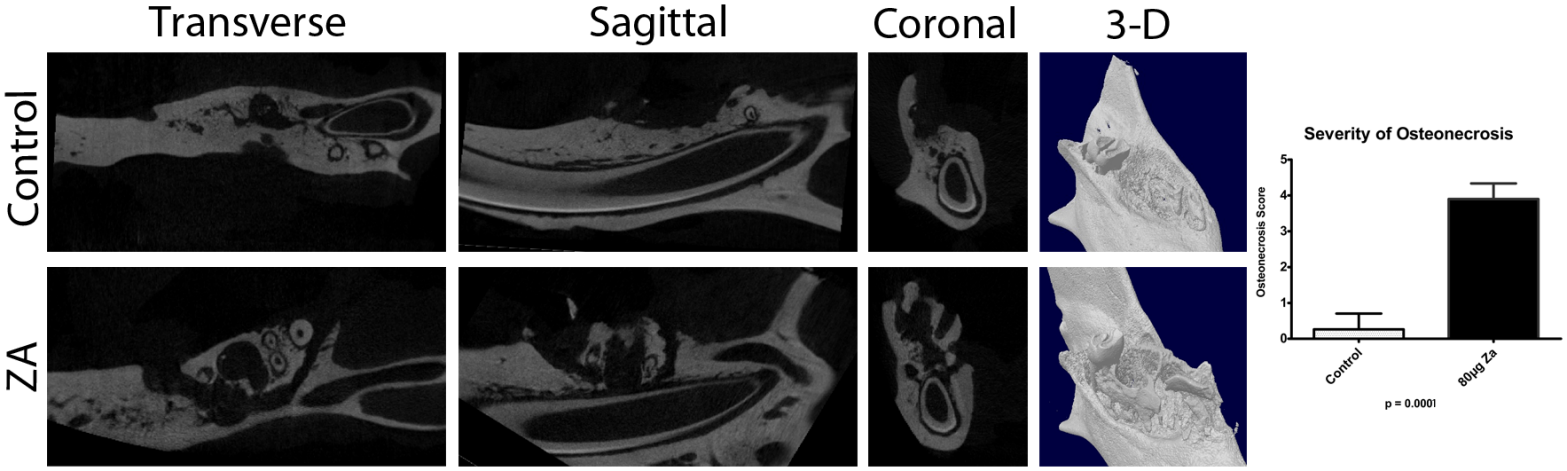
MicroCT images of Control and ZA-treated mandibles, eight weeks after extractions, showing massive sequestration in a treated animal, compared to normal healing in the control. Quantifying the severity of osteonecrosis on a 0–4 scale, the ZA-treated animals received a score between 3 and 4, compared to 0–1 in control animals.

Control animals (Fig 2 and S1 Fig) showed normal socket healing, with uniform new bone extending from the base and sides to fill the socket cavity, and extruding any remaining root tips towards the surface. In contrast, all ZA-treated animals (Fig 2 and S2 Fig) showed lack of bone formation within the socket, in addition to substantial fragmentation of the alveolar bone, extending from the extraction site. Large blocks of sequestered bone were consistently evident in multiple planes, with fissures and fracture lines scattered throughout the remaining alveolar bone.

### Severity of BRONJ

At the 8^th^ week, five out of 20 control animals had remaining areas of mucosal dehiscence either because of an exposed root tip within the extraction site, which were confirmed with micro-CT, or mucosal damage, which could be due to impacted food particles or repeated trauma from the opposing tooth. In these animals, there was no evidence of sequestered bone was found on microCT; therefore, they received a BRONJ severity score of 1. One control animal died because of anesthesia-related complication during the week 2 follow up examination, while the remaining 14 animals had complete mucosal coverage and no sequestration thus receiving a score of 0. In contrast, 19 of the treatment animals received a score of 4, because they lacked soft tissue coverage over large areas of necrotic bone, with consistent micro-CT evidence of bone sequestration of bone in all 3 planes and massive destruction of alveolar bone beyond the extraction site. The remaining animal received a score of 2 because it showed mucosal dehiscence overlying necrotic bone, with micro-CT evidence of sequestered bone in 2 of the 3 planes. The treated animals had a much higher severity of BRONJ than the control (3.9±0.435 versus 0.26±0.44, respectively; p<0.001) (Fig 2).

### Histological Evaluation of Bone Healing

**Fig 3:**
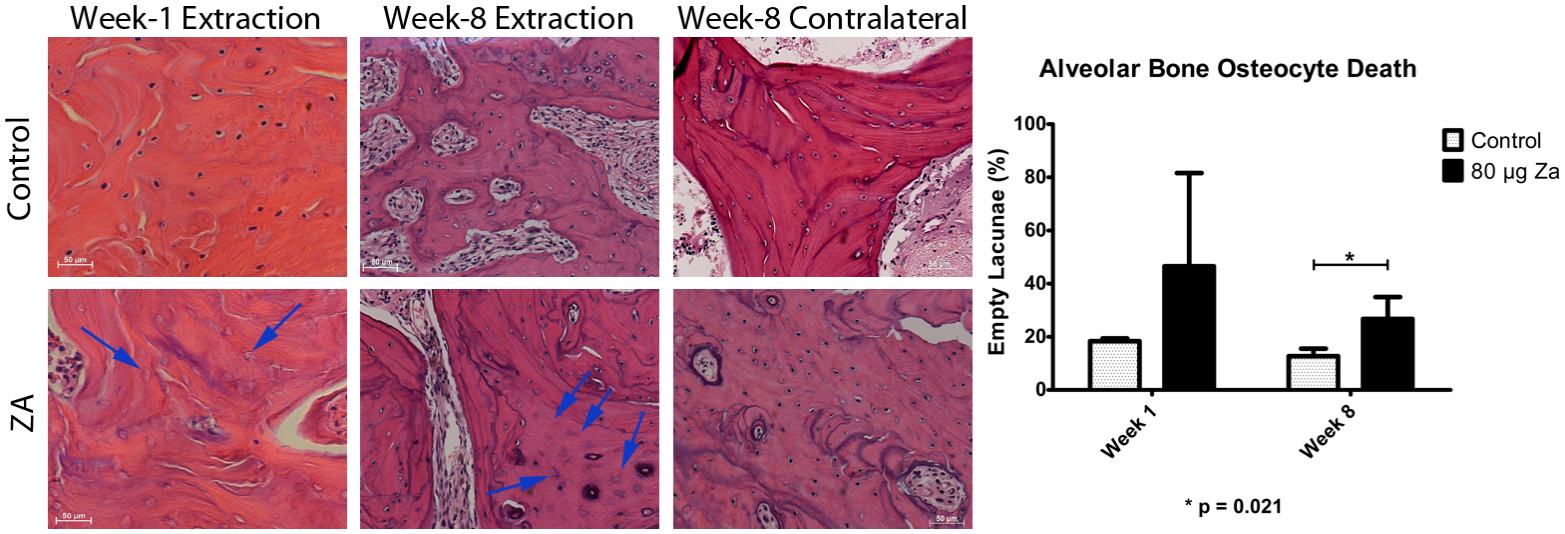
Analysis of alveolar bone at the extraction site, excluding any sequestered bone. The numbers of empty lacunae over total number of osteocyte lacunae were used to calculate the percentage of dead osteocytes within the bone. There was a significant increase in the percentage of empty lacunae (arrows) on the extraction sites on ZA-treated animals. There was also a significant decline in the numbers of empty lacunae in the control group from week 1 to week 8. The un-operated sites in the ZA-treated animals did not show a significant difference in bone viability, compared to the un-operated sites in control animals, signifying that surgical extraction was necessary to illicit bone necrosis.

Evaluation of H&E stained sections of the extraction site was carried out at 1 and 8 weeks after extraction, excluding the bone sequestra (Fig 3). The percentage of empty lacunae within the remaining walls of the socket at 1-week was higher, although not significantly, in the treatment group (46.527±35.1%) than the control (18.346±1.04%) (p = 0.377). At 8-weeks, the treatment group also had significantly higher percentage of empty lacunae (26.73±8.21%), than the control animals (12.68±2.87%) (p = 0.021). Within the ZA-treated animals, the operated side had much higher percentages of empty lacunae than the un-operated (contralateral) sides (15.45 ± 9.39%) (p<0.001). There was no difference on the un-operated sites between the control (14.44 ± 5.79%) and treated (15.45 ± 9.39%) animals (p = 0.327). There was also no detectable bacterial colonization in the bone at the extraction sites in the control or ZA-treated animals one week after extraction.

### Dynamic Parameters

**Fig 4:**
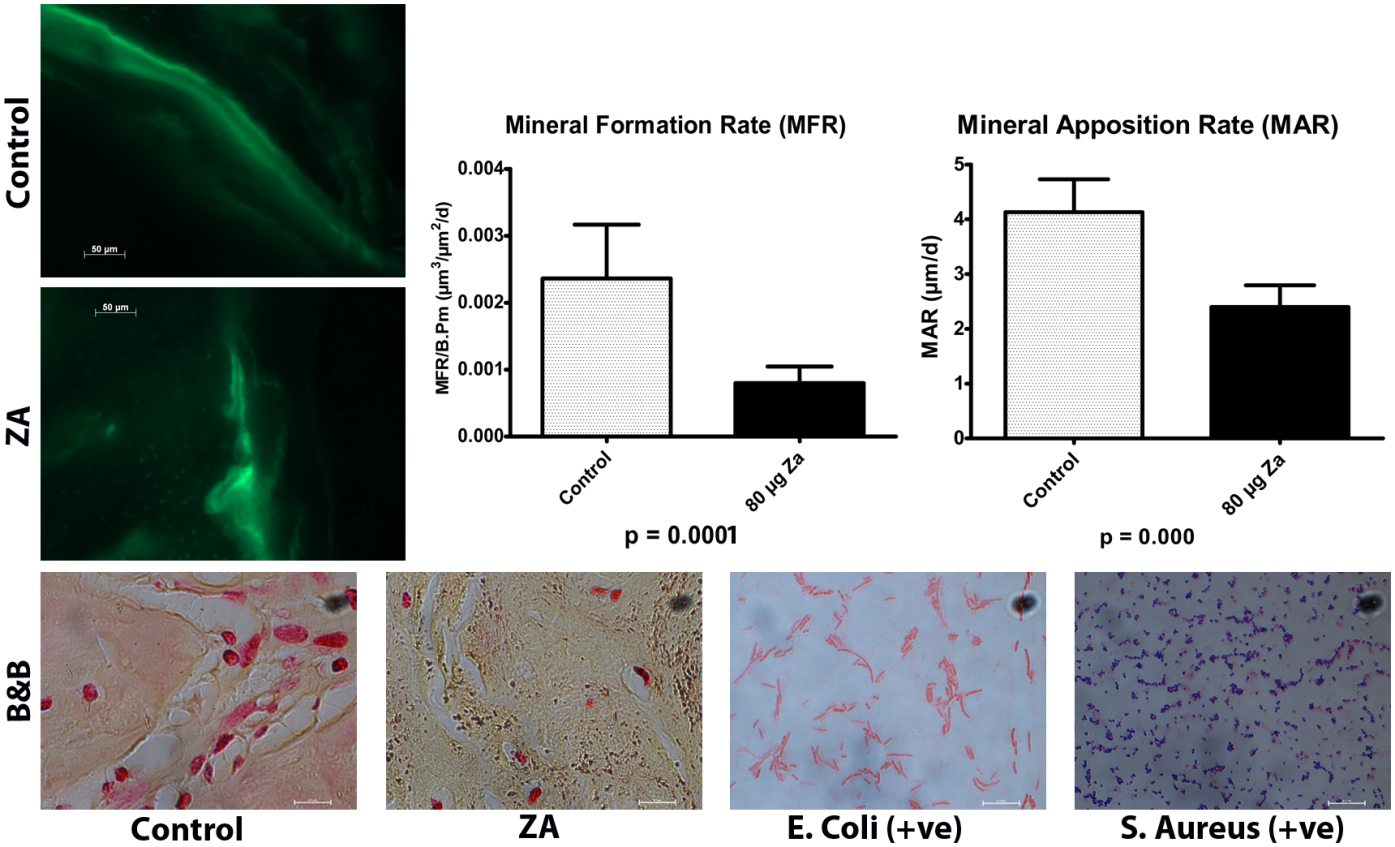
Calcein double-labeling in Control and Treatment groups within the extraction sites have shown that Mineral Formation Rate (MFR) and mineral Apposition Rate (MAR) within the extraction sites 2 weeks post extraction was diminished in the ZA-treated animals, compared to controls. Bottom panel: Staining with modified Brown and Brenn (B&B; 100x) stain showed minimal to no bacterial colonization of alveolar bone either in control or ZA-treated animals.

The mineral apposition rate (MAR) was significantly impaired in the treatment group (2.40±0.39μ/day), compared to the control (4.13±0.60μ/day), by a two-tailed student’s t-test (p<0.001; Fig 4). Mineral formation rate (MFR) was significantly impaired in the treated group (0.79E-3±0.24E-3 μ^2^/day) than the control (2.36E-3±0.80E-3) (p<0.001).

### Osteoclast Activity

**Fig 5:**
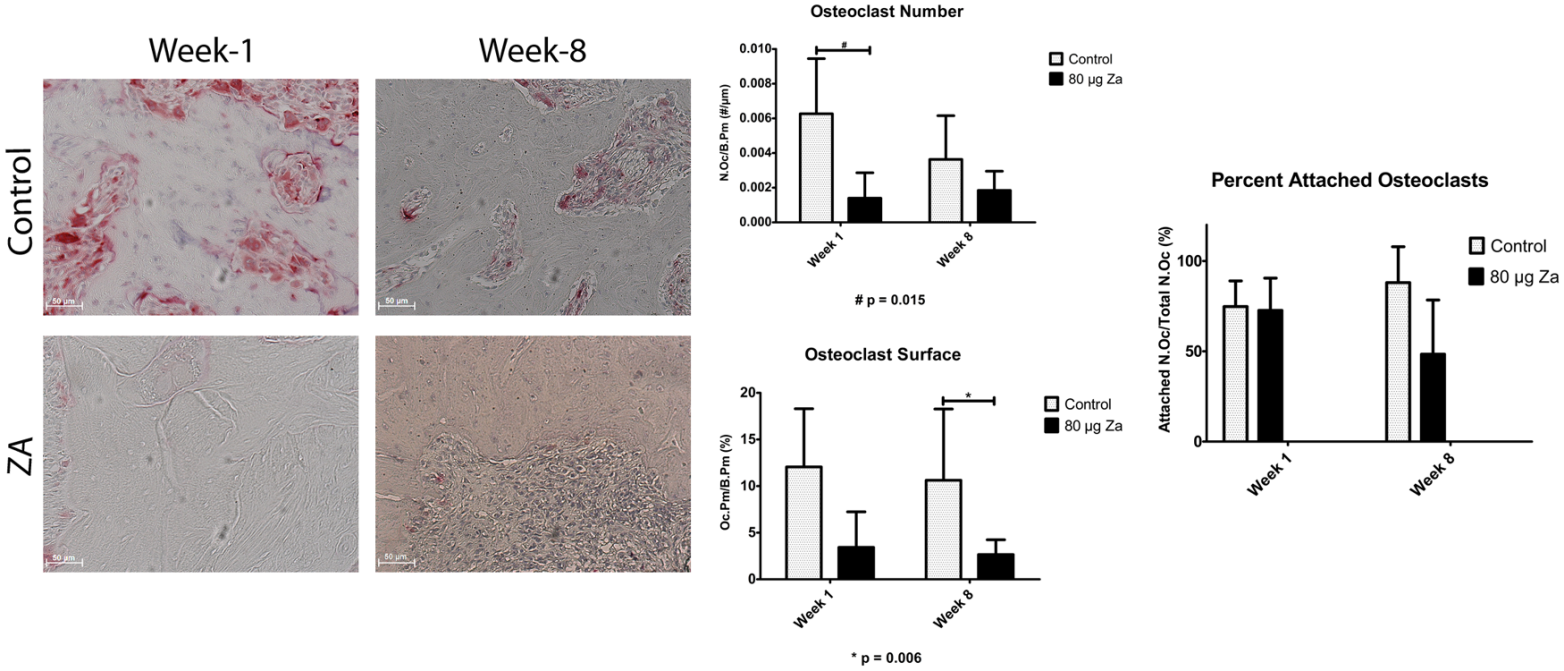
TRAP-labeled decalcified sections from dental extraction sites at 1-week following extraction. A. Characterization of the number of Oc (N.Oc) normalized to the bone surface perimeter (B.Pm). B. Osteoclast perimeter vs bone surface perimeter.

At 1-week, the osteoclast number (N.Oc/B.Pm) was significantly lower in the treatment group (1.39E-3±1.47–3μ^-1^) then the control (6.26E-3±3.18E-3 μ^-1^) (p = 0.015; Fig 5). At 8 weeks, however, there was no significant difference between the control (3.63E-3±2.52E-3 μ^-1^) and treated (1.84E-3±1.11E μ^-1^) groups (p = 0.565). Osteoclast surface was lower, albeit not significantly, in the treated (3.4±3.8%) than the control group (12.06±6.2%) at week 1 (p = 1.000); and significantly lower in the treated (2.66±1.6%) than the control group (10.6±7.6%) at week 8 (p = 0.006). Osteoclast surface significantly increased in the control group between 1 and 8 weeks (p = 0.003), but did not change in the treated group between the two time points (p = 1.000). The percentage of attached osteoclasts showed a strong, but not statistically significant (p = 0.068), decline in the treatment group between 1 and 8 weeks, compared to the control. Taken together, osteoclast recruitment was reduced in the treated animals at 1-week. At week-8 osteoclast recruitment partially recovered; however, osteoclasts failed to attach to the bone surface or became detached after initial attachment.

### Liver and Kidney Histopathology and Function

**Fig 6:**
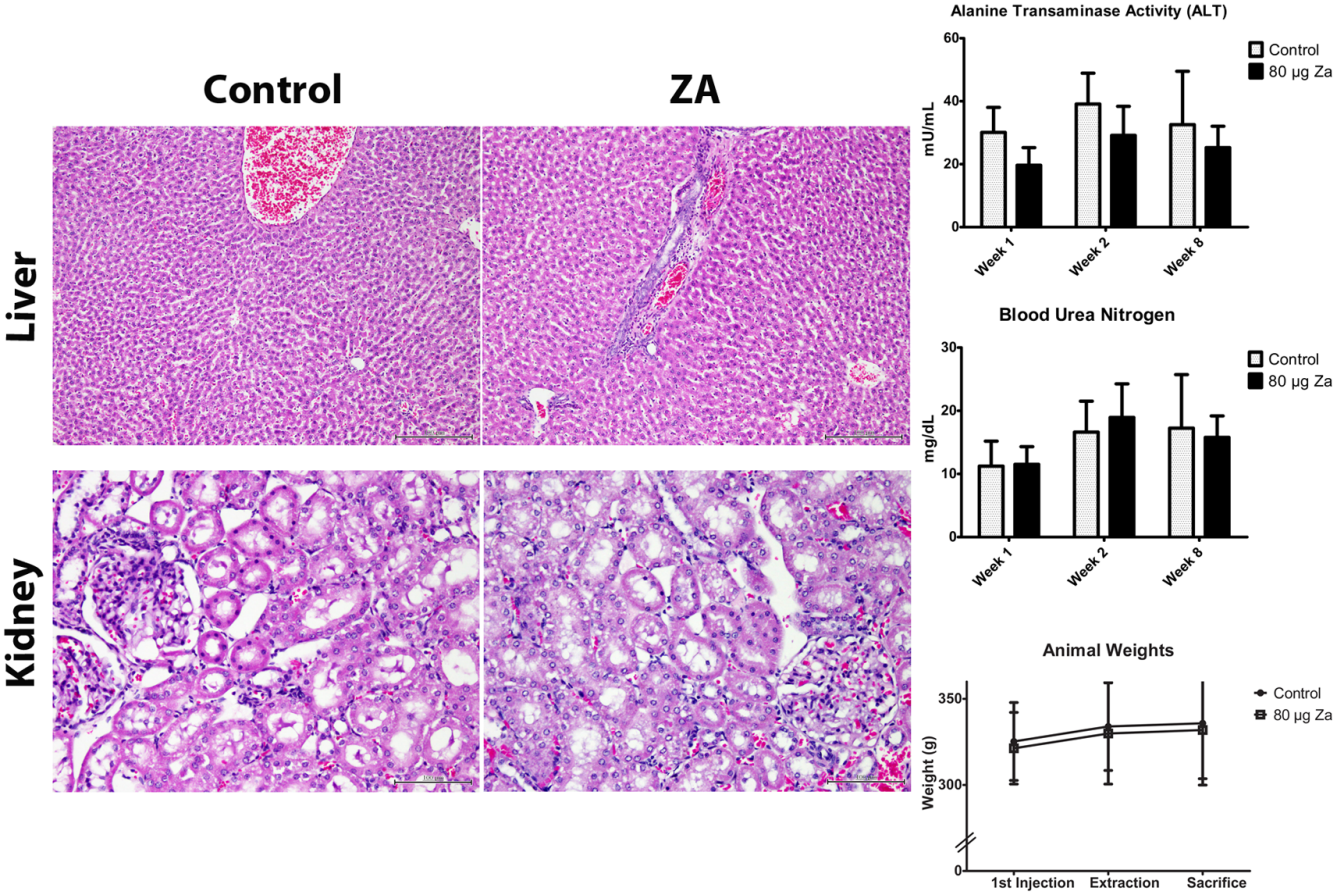
Systemic effects of ZA treatment. A: No significant difference in kidney histology or BUN serum level between control and ZA-treated animals. 9B: No significant difference in liver histology or ALT activity, used to evaluate liver function between the two groups. 9C: No significant difference in body weights between the two groups.

Kidney sections exhibited mostly intact glomeruli, and scattered swelling, occasional apoptosis and sloughing of tubular cells, which were similar in the control and treatment groups. Scattered inflammatory infiltrates were sometimes present in both groups. Morphologic abnormalities in 2-week samples appeared mainly related to inadequate tissue fixation. Liver sections exhibited normal architecture. There were mild perivascular infiltrates of chronic inflammatory cells, scattered apoptotic cells, and dilated sinusoids, irrespective of the group. Fixation defects prevented exhaustive assessment of the morphologic features; so kidney and liver function tests were performed. No differences were detected in BUN levels between treatment and control groups at the 1, 2, and 8-week time points (Fig 6). Similar findings were evident for the ALT levels, at each of the time points, used to test liver function (Fig 6). Both the control and treatment groups gained weight throughout the duration of the experiment, with no difference between the groups (p = 0.647).

## Discussion

The aim of the current study was to test a BRONJ model with the following characteristics: 1) demonstrated consistent occurrence of the full features of BRONJ in bisphosphonate-treated animals, using repeated surgical extractions; 2) excluded the systemic toxic effects of the bisphosphonate regimen on the liver and kidney; and 3) provided a quantitative BRONJ analysis. We hypothesized that repeated surgical extraction (major trauma) would be sufficient to induce consistent, severe form of BRONJ in rats on long-term zoledronate therapy, without the need to induce pre-existing dental and/or systemic pathology.

All zoledronate-treated animal developed the gross, radiologic, and histological features of severe BRONJ for 8 weeks after extraction, fulfilling the clinical definition of severe BRONJ.[21] Most treated animals (19/20) were classified as grade 4-severity on a 0–4 scale. Importantly, the lack of zoledronate-related organ toxicity or independent systemic pathology suggests that the bone necrosis is likely due to a direct effect of the drug on bone.

At 1-week, osteoclast number was lower in the ZA-treated animals, which could signify defective osteoclast recruitment and attachment. Such an effect on osteoclasts is shared with other anti-resorptive drugs.[4,22] After 8-weeks of stopping the treatment, osteoclast number recruitment partially improved. However, osteoclast attachment continued to be severely defective in the treated group. Even at the un-sequestered bone, which remained relatively viable, osteoclasts attachment continued to be deficient. This finding may be explained by the fact that, unlike other anti-resorptive drugs, bisphosphonates physically accumulate in alveolar bone,[23] suggesting possible involvement of local mechanisms in BRONJ pathogenesis, in addition to the short-term systemic effect on osteoclasts, which is common to all anti-resorptive drugs.

As the treatment continues, both larger amounts of zoledronate accumulate and larger areas of bone matrix become involved.[24] Indeed, the higher the affinity of bisphosphonate to bone, the more potent it is.[25] When dental extraction occurs, the first-responding osteoclasts internalize zoledronate, leading to their detachment and apoptosis, halting the healing process, with subsequent bacterial colonization and progressive osteonecrosis.[26] It is reasonable to assume, therefore, that bone remodeling would not effectively start except at an intermediate zone, where enough zoledronate-free surfaces are available for osteoclasts to attach and start separating the viable bone from the dead (sequestration).

Our histomorphometric findings confirmed the osteonecrosis observed clinically and rediographically in ZA-treated animals. The treated animals showed massive areas of sequestered bone. Even the remaining (un-sequestered) areas still showed decreased vitality in ZA-treated animals, compared to contralateral bone and to extraction sites in un-treated animals. The contralateral (un-operated) side in ZA-treated animals did not show any osteonecrosis, when compared to the un-operated sides of the untreated animals, confirming that dental trauma was a necessary triggering event for BRONJ in this model. Also, there was no detectable bacterial colonization one week after extraction in either the control or ZA-treated animals, suggesting that dental extraction by itself was sufficient to elicit the osteonecrosis. Based on these finding, the repeated surgical extraction was sufficient to consistently induce BRONJ in rats. Future studies are underway to explore the role of oral microorganisms in the induction and progress of BRONJ in this model.

Dynamic bone analysis confirmed the diminished bone healing within the socket in ZA-treated animals. Bone formation was impaired in treated animals, in addition to the expected impairment in bone resoption. There has been strong evidence in the literature that bisphosphonate therapy does not impair bone healing after invasive trauma at bone sites other than the jaw.[27] However, the dental socket has been proven an exception, showing signs of impaired regeneration after dental extraction.[28–30] The underlying mechanisms explaining such a discrepancy in post-traumatic healing at different bone sites are not yet understood.

To date, no direct causal link has been established between bisphosphonate treatment and the occurrence of BRONJ.[31] Spontaneous osteonecrosis of the jaw still occurs in patients who are not receiving bisphosphonates;[1] and there is an ongoing debate on whether the rate of BRONJ with oral bisphosphonates is not higher than that of spontaneous osteonecrosis of the jaw in the same population.[8] However, it is now widely accepted that long-term intravenous bisphosphonate therapy carries a much higher risk of osteonecrosis of the jaw; and that such risk is highest after dental extraction.[8] Therefore, a good place to start the quest for a causal relationship would be to model these conditions in a reproducible animal model.[1]

Although multiple studies reported the occurrence of BRONJ-like features in animal models, success rates and reproducibility have been highly variable (Table 1). Multiple studies used supra-therapeutic dose regiments.[5,15,32–34] In these studies, it could be argued that the bone necrosis might be a toxic effect of drug overdose, especially where no data were provided about kidney or renal functions in treated animals. Other studies used massive oral trauma[7,35–37] to illicit the condition. For example, while Pautke and co-investigators observed a 80% incident rate of BRONJ in the treatment group, it was only inducible after extracting three maxillary and three mandibular molars in a single surgical setting.[35] Our model proved that repeated surgical extraction was sufficient to illicit severe BRONJ in 100% of animals. Other studies demonstrated osteonecrosis at earlier time points than the eight-week cut-off outlined for the clinical definition of BRONJ,[15,34,36–39] or restricted the evidence of BRONJ to its clinical presentation of exposed bone, without radiographic or histological evidence of osteonecrosis.[3,5,10,39]

More studies demonstrated that BRONJ-like lesions developed in vitamin D-deficient rats[6] or rats on dexamethasone.[7] In these studies, however, rats treated with bisphosphonates alone did not develop the disease, suggesting that the BRONJ features in these animals were induced via mechanisms related to the pre-existing comorbidity. The most promising results were produced using periodontitis and periapical lesions.[2,3] It may be true that in human patients who undergo dental extraction, the procedure may have been indicated by an active infection. However, in these cases, BRONJ mostly manifested after surgery. It was important to test whether trauma without pre-existing inflammation and/or infection would be sufficient to induce BRONJ. It can be argued, however, that the first extraction is a pre-existing comorbidity at the time of the second extraction. Nonetheless, the inducing factor for BRONJ remains to be the repeated surgical procedure with no need to use an independent pathology that could be difficult to calibrate. We have observed no sign of active infection at the extraction site at the time of the second extraction, a finding confirmed by bacterial staining. Furthermore, the model was simple, precise, and with a predictable rate of BRONJ induction.

We believe that consistent induction of the complete manifestation of BRONJ requires repeated surgical extractions. In our experience, simple dental extraction (without drilling) and/or the use of a single procedure would induce variable degrees of BRONJ-like lesions that would be inconsistent between animals.[9,10] Comparing group parameters, as opposed to an animal-by-animal analysis, would not have the best translational value to the human condition. The model presented here allows the utilization of both the incidence and severity of BRONJ as dependent variables.

Overall, the rate of induction of BRONJ in previous studies varied from 0% [29,32] to a 100%, making it very difficult to use these models to establish causality.[40] At least some of this variability could be due to the difficulty in calibrating the inducible factors, such as periodontitis and peri-apical leasions, or the variability in dose regimens of bisphosphonates. The dose we used in this study was 21% higher than the clinical dose (80 μg/kg versus 66 μg/kg body weight). However, it remained well within the safety margin of zoledronate.[24] In human patients, the overall prevalence of BRONJ with zoledronate increased with the duration of treatment.[8,14] The mean time to BRONJ development after the start of ZA treatment was 1.8 years (minimum 10 months).[41] The remodeling cycle in rats is 20 times shorter than humans.[42] Accordingly, the 13-week treatment is comparable to 4.6 years in humans.

Finally, we focused our studies on female animals for the following reasons: 1) to be able to compare the results to our earlier studies that used the osteoporosis dose of zoledronate and 2) because of the descripency in male versus female rates regarding size and weight at the same age. For a baseline study, we decided to continue using only female rats to establish the experimental procedure that would precipitate BRONJ in all animals. In an ongoing larger study, we are including equal numbers of male and female rats.

Despite the debilitating nature of BRONJ, no effective treatment strategies have been developed to date.[31] The only effective prevention strategy seems to be to avoid dental procedures in patients on long-term intravenous bisphosphonates altogether.[1] Our model is unique because it fulfilled the following three necessary requirements for a translational model suitable for prevention or therapeutic studies: first, it demonstrated consistent and reproducible occurrence of the full characteristics of BRONJ in treated animals using repeated major trauma (surgical extraction); second, it excluded the systemic toxic effects of the zoledronate regimen that could confound the bone effect; and third, the BRONJ characteristics could be quantified into stages. Our results provide the basis for subsequent studies isolating the primary mechanism of BRONJ as well as testing methods for preventing BRONJ. Studies are currently underway in this direction.

## Supporting Information

S1 Fig Images from three representative control (untreated) animals at 8 weeks after the second surgical extraction. Each set shows the extraction site (top left) and three micro-CT sections at the extraction site (sagittal, coronal, and axial) to detect bone sequestration. Control animals showed adequate mucosal healing, while micro-CT revealed smooth bone regenerate in the extraction site with no sequestration.
(TIF)

S2 Fig Images from three representative ZA-treated animals at 8 weeks after the second surgical extraction. Each set shows the extraction site (top left) and three micro-CT sections at the extraction site (sagittal, coronal, and axial) to detect bone sequestration. The mucosa overlying the extraction site failed to heal, revealing necrotic bone underneath. Micro-CT revealed massive fragmentation (sequestration), with the alveolar bone separated from the rest of the mandible in all three planes.
(TIF)
