# Supplementary figures and images for: A Model for Osteonecrosis of the Jaw with Zoledronate Treatment following Repeated Major Trauma
Source: PLoS One. 2015 Jul 17;10(7):e0132520. doi: 10.1371/journal.pone.0132520 (PMC4505856; doi:10.1371/journal.pone.0132520)

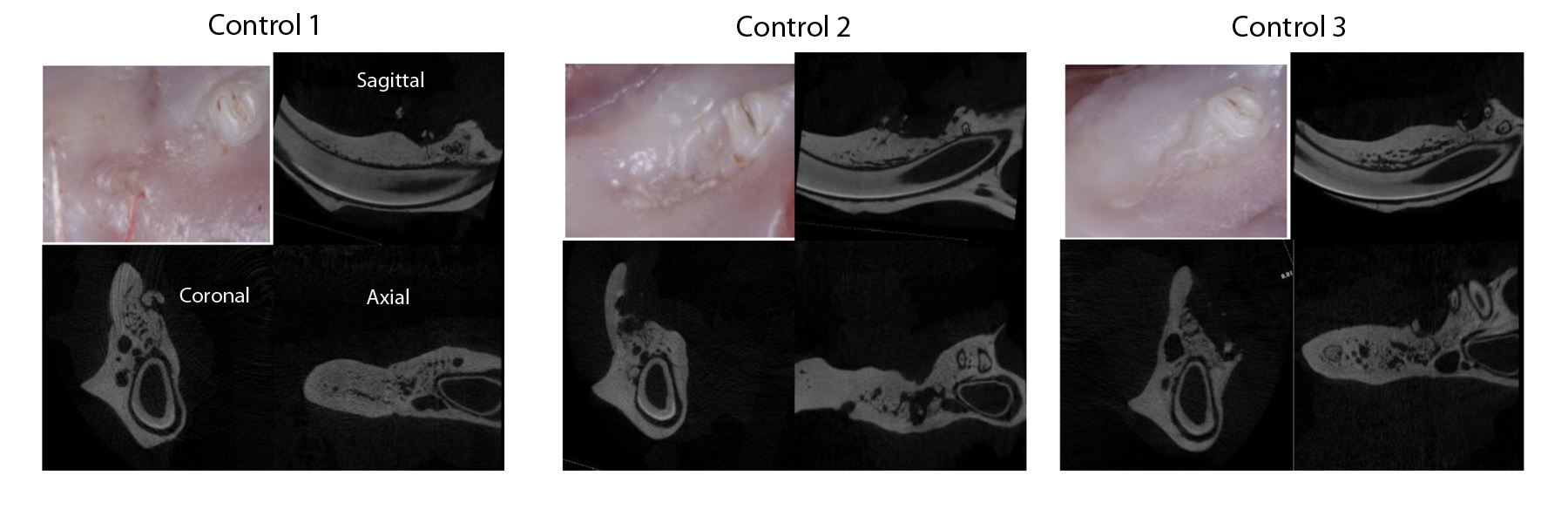

Supplement: S1 Fig — Each set shows the extraction site (top left) and three micro-CT sections at the extraction site (sagittal, coronal, and axial) to detect bone sequestration. Control animals showed adequate mucosal healing, while micro-CT revealed smooth bone regenerate in the extraction site with no sequestration. (TIF) [file pone.0132520.s001.tif]

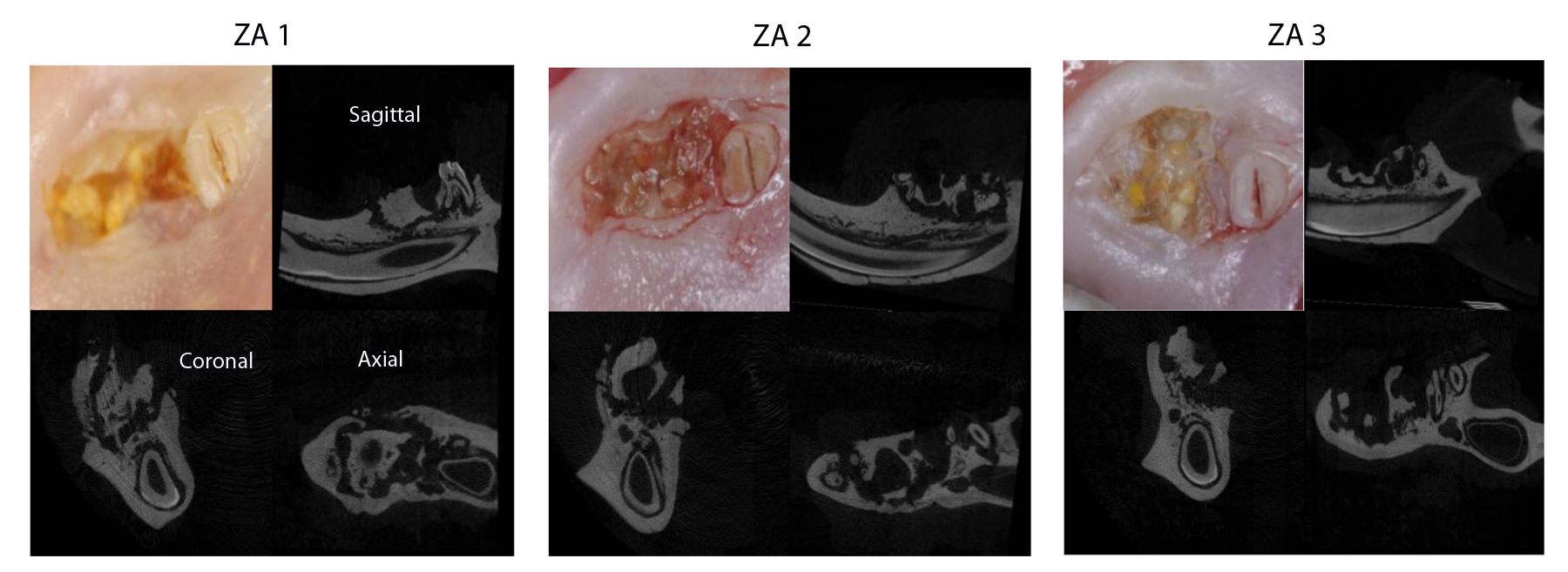

Supplement: S2 Fig — Each set shows the extraction site (top left) and three micro-CT sections at the extraction site (sagittal, coronal, and axial) to detect bone sequestration. The mucosa overlying the extraction site failed to heal, revealing necrotic bone underneath. Micro-CT revealed massive fragmentation (sequestration), with the alveolar bone separated from the rest of the mandible in all three planes. (TIF) [file pone.0132520.s002.tif]
